# Do seasonal patterns of rat snake (*Pantherophis obsoletus*) and black racer (*Coluber constrictor*) activity predict avian nest predation?

**DOI:** 10.1002/ece3.1992

**Published:** 2016-02-26

**Authors:** Brett A. DeGregorio, Patrick J. Weatherhead, Michael P. Ward, Jinelle H. Sperry

**Affiliations:** ^1^ Department of Natural Resources and Environmental SciencesUniversity of IllinoisChampaign‐UrbanaIllinois; ^2^ Engineer Research and Development CenterChampaignIllinois

**Keywords:** Activity patterns, automated radiotelemetry, nest cameras, nest survival, northern cardinal, predator–prey

## Abstract

Avian nest success often varies seasonally and because predation is the primary cause of nest failure, seasonal variation in predator activity has been hypothesized to explain seasonal variation in nest success. Despite the fact that nest predator communities are often diverse, recent evidence from studies of snakes that are nest predators has lent some support to the link between snake activity and nest predation. However, the strength of the relationship has varied among studies. Explaining this variation is difficult, because none of these studies directly identified nest predators, the link between predator activity and nest survival was inferred. To address this knowledge gap, we examined seasonal variation in daily survival rates of 463 bird nests (of 17 bird species) and used cameras to document predator identity at 137 nests. We simultaneously quantified seasonal activity patterns of two local snake species (*N* = 30 individuals) using manual (2136 snake locations) and automated (89,165 movements detected) radiotelemetry. Rat snakes (*Pantherophis obsoletus*), the dominant snake predator at the site (~28% of observed nest predations), were most active in late May and early June, a pattern reported elsewhere for this species. When analyzing all monitored nests, we found no link between nest predation and seasonal activity of rat snakes. When analyzing only nests with known predator identities (filmed nests), however, we found that rat snakes were more likely to prey on nests during periods when they were moving the greatest distances. Similarly, analyses of all monitored nests indicated that nest survival was not linked to racer activity patterns, but racer‐specific predation (*N* = 17 nests) of filmed nests was higher when racers were moving the greatest distances. Our results suggest that the activity of predators may be associated with higher predation rates by those predators, but that those effects can be difficult to detect when nest predator communities are diverse and predator identities are not known. Additionally, our results suggest that hand‐tracking of snakes provides a reliable indicator of predator activity that may be more indicative of foraging behavior than movement frequency provided by automated telemetry systems.

## Introduction

Avian nest success often varies seasonally and spatially (Perrins 1970; Price et al. 1988), and because predation is the primary cause of nest failure for many birds (Martin 1993), variation in predator abundance or behavior has been hypothesized to explain patterns of nest success (Heske et al. 1999; Burhans et al. 2002; Chalfoun et al. 2002). Nest predators and parasites are typically not evenly distributed throughout landscapes (e.g., Andren 1992; Rich et al. 1994; Chalfoun et al. 2002), and some studies have made strong connections between the behavior of predator species and avian nest survival. Klug et al. (2010) showed that two species of snake preferentially use shrub within grasslands and that avian nest survival was lower in this habitat. Similarly, DeGregorio et al. (2014a) found that avian predators were most often encountered along power line right‐of‐ways and nests along these corridors were more likely to be preyed on by these predators. However, in other cases, no direct relationships between predators and nest survival were found. Sperry et al. (2009) showed that Texas rat snakes (*Pantherophis obsoletus*) preferred locations with more structure and closer to cover objects or edges but these habitat characteristics did not influence nest survival of two focal bird species. Similarly, raccoons (*Procyon lotor*) are often associated with edges in fragmented landscapes but these patterns did not correlate with avian nest survival rates (Heske et al. 1999). Identifying relationships between predator activity and nest predation may be challenging when the nest predator community is diverse (Klug et al. 2009). In some areas, however, particular species or guilds can be important predators (e.g., snakes in the southeast: DeGregorio et al. 2014b). Despite varying results, understanding patterns in predator behavior in relation to nest survival holds promise for better understanding patterns in avian reproductive success and nesting ecology.

The reported links between predator behavior and avian nesting success have come from studies of seasonal patterns of predator activity, primarily but not exclusively snakes, and nest survival. Snakes can be regionally important nest predators (Weatherhead and Blouin‐Demers 2004; Reidy and Thompson 2012; DeGregorio et al. 2014b), and snakes often exhibit substantial seasonal variation in activity (Gibbons and Semlitsch 1987; Bernardino and Dalrymple 1992; Abom et al. 2012). Seasonal variation in snake activity is attributed to patterns in reproductive cycles and seasonal variation in temperature (Moore 1978; Shine 1979; Gibbons and Semlitsch 1987; Abom et al. 2012). Three studies have shown an association between seasonal patterns in snake activity and nest success of syntopic birds (Sperry et al. 2008, 2012; Weatherhead et al. 2010). However, the link between snake activity and nest success has been shown to vary between bird species and between studies. For instance, the link between nest predation of the black‐capped vireo (*Vireo atricapilla*) and snake activity was strong at a site in Texas, but the association between nest success and snake activity was less pronounced for golden‐cheeked warblers (*Dendroica chrysoparia*) at the same site (Sperry et al. 2008). Similarly, Weatherhead et al. (2010) showed a link between the activity of rat snakes and nest survival of blue‐gray gnatcatchers (*Polioptila caerulea*), field sparrows (*Spizella pusilla*), and northern cardinals (*Cardinalis cardinalis*) but not for yellow‐breasted chats (*Icteria virens*), indigo buntings (*Passerina cyanea*), or Acadian flycatchers (*Empidonax virescens*). The inconsistency of these results raises questions about the generality of this phenomenon and may be a consequence of none of the studies having been designed to identify predators at bird nests. Instead, snake activity and nest success were both studied at the same locations and any link between them was inferred. A much stronger approach would involve identification of the predators responsible for nest predation so that analyses could be restricted to nests preyed on by a particular predator species and seasonal variation in that species' activity and thus not be confounded by the effect of other nest predators.

If more active snakes find more nests, seasonal increases in snake activity should increase nest predation rates. The most reliable and repeatable method to quantify snake activity is radiotelemetry, although this method is not without its shortcomings. To date, snake activity has been quantified using manual radiotelemetry where snakes are located between one and five times a week, and the distance moved between successive locations is assumed to be linear. Incomplete sampling associated with this method is likely to provide an incomplete record of snake activity. Automated radiotelemetry continuously monitors snake activity (Kays et al. 2011) and allows quantification of the frequency of movements by an individual to complement the distance moved per day calculated by manual radiotelemetry. Additionally, nest cameras allow researchers to reliably identify nest predators (e.g., Cox et al. 2012a). Here, we combine manual and automated radiotelemetry of two snake species with video surveillance of bird nests to examine links between seasonal snake activity patterns and snake‐specific nest predation rates.

**Figure 1:**
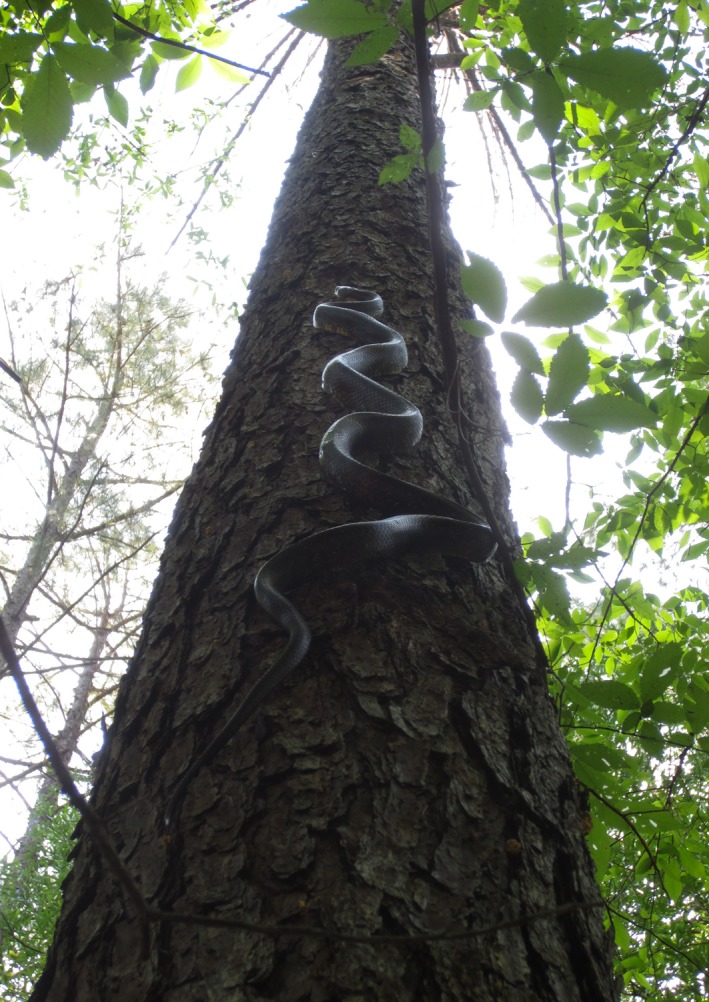
The rat snake (*Pantherophis obsoletus*) is a major predator of bird nests throughout much of the southeastern and central United States. Photograph by Patrick Roberts.

Twelve species of North American snakes have been identified as predators of bird nests (DeGregorio et al. 2014b), and these species vary considerably in their ecology and behavior. The black racer (*Coluber constrictor:* hereafter, racer) is a fast‐moving, diurnal species that opportunistically pursues prey encountered as it moves through the environment (Ernst and Ernst 2003). Although racers are common predators of bird nests (e.g., Thompson et al. 1999; Thompson and Burhans 2003; Klug et al. 2009), they are often relatively minor contributors to overall nest predation (11–16%: Thompson et al. 2003; Klug et al. 2009) at particular sites (DeGregorio et al. 2014b). The one study that examined seasonal patterns of racer activity and nest predation found an association for only one of six focal nesting species (Acadian flycatcher: Weatherhead et al. 2010). However, the habitat use of racers has been linked to increased predation risk for grassland birds (Klug et al. 2009). Conversely, the rat snake (*Pantherophis obsoletus*) (Fig. 1) can be an important nest predator, contributing to substantial nest failure within a system (28–40%: Thompson et al. 1999; Carter et al. 2007; Reidy and Thompson 2012), including at our study site (28%: DeGregorio et al. 2014a). Rat snake activity varies seasonally, with minimal activity early in the spring and late in the summer and peaks in activity in May and June (e.g., Carfagno et al. 2008; Sperry et al. 2008, 2012). In three studies, rat snake activity mirrored patterns of nest predation for black‐capped vireos (*Vireo atricapilla*), blue‐gray gnatcatchers, field sparrows, and northern cardinals (Sperry et al. 2008, 2012; Weatherhead et al. 2010).

Our goals here were to (1) compare the snake activity patterns between automated and manual radiotelemetry, (2) evaluate whether snake activity is correlated with overall avian nest success, and (3) evaluate if snake activity is correlated with predator‐specific predation rates determined via nest cameras.

## Methods

### Study site

Our study took place at the U.S. Department of Energy's Savannah River Site in South Carolina. Specifically, we worked at Ellenton Bay Set Aside Research Area. The site is mostly forested with mixed forests aged 50–70 years. Although forested, the site is highly fragmented with clear‐cuts, utility right‐of‐ways, dirt roads, and open shrubland patches. Located in the south‐central portion of the site is Ellenton Bay, at 10 ha Carolina bay wetland. We focused our nest monitoring efforts on an approximately 200 ha area of the Set Aside Research Area. We focused snake capture for telemetry on this same area, although subsequent snake movement resulted in our radiotelemetry efforts occurring over an area of approximately 800 ha (Appendix S1).

### Snake behavior

We used radiotelemetry (both manual and automatic) to assess the seasonal activity patterns of rat snakes and racers. We manually tracked individuals of each species from March to August of 2011–2013 and used continuous automated radiotelemetry during the same months of 2012 and 2013. We primarily relied on opportunistic captures of snakes during the nesting seasons. We transported all captured snakes to a veterinarian for transmitter implantation surgery following the protocol of Blouin‐Demers and Weatherhead (2001). Transmitters (model SI‐2T 9 g, 11 g, or 13 g, Holohil Systems Ltd., Carp, ON, Canada) weighed <3% of the snake's total mass. We should note that racers were most frequently associated with open habitats but did occur in all available habitat types (including the wetland). Rat snakes were most frequently located in forested habitats, but also used all available habitats. We released snakes at their capture spot after 3–5 days postsurgery. We hand‐tracked snakes throughout the day and night at approximately 2‐day interval and marked each location with handheld GPS units. We tracked each individual at least 3–4 times per week but the tracking interval often changed due to weather conditions. To quantify seasonal patterns in activity from hand‐tracking data, we followed the standard protocol of calculating “distance moved per day” (e.g., Reinert and Kodrich 1982; Macartney et al. 1988). Using GIS software (Arcmap 10.0; ESRI Inc., Redlands, CA), we calculated the distance between successive snake relocations and divided by the number of days between relocations. Then, we averaged this value across all snakes for 1‐week interval to calculate seasonal trends in daily distance moved per day for each snake species. Although this is the most frequently reported metric of snake activity in the literature, this estimate of distance moved per day may underestimate snake movement because it assumes that snakes move from point‐to‐point in a linear fashion (Ward et al. 2013).

We used automated radiotelemetry (Automated Receiving Units: ARUs: JDJDC Corp., Fisher, IL) to complement hand‐tracking. ARUs provided detailed data regarding the movements of snakes. We connected each receiving unit to six Yagi antennas attached via a hub on a 10‐m tower. We positioned the four towers in a rough diamond pattern specifically configured to maximize the number of transmittered animals that could be detected. We spaced the six antennas by 60° to give 360^°^ coverage. Once the tower was assembled, we then programmed each ARU to tune at 5‐ to 15‐min interval (rapid tuning when few snakes were being tracked and slower tuning when many snakes were being tracked) to each transmitter frequency. At each interval, the ARUs recorded the signal strengths (in dBm) and bearing from the six antennas to each snake. We downloaded data from the ARUs approximately every 12–14 days during the nesting season.

Determining snake activity using ARUs involves detecting changes in both bearing and signal strength. By moving around a test transmitter, we were able to conclude that a snake was moving if its bearing changed by 2° in and its strength changed by 150 dBm between successive readings. Using these thresholds, we were able to detect relatively small snake movements (<2 m) reflective of snakes exploring and moving within habitat features (logs, brushpiles, snags). Before data analysis, we filtered the data to eliminate spurious and weak records using the protocol described by Ward et al. (2013).

To evaluate seasonal trends in snake movement from automated radiotelemetry data, we quantified the total number of snake detections for each individual of each species and the proportion of those detections in which the snake was actively moving. We then separated these data into 1‐week period from the start to the end of the avian nesting season and calculated the proportion of detections each week that each individual was active. Thus, if we had 2000 rat snake detections during the first week of April and snakes were moving during 140 of those detections, we determined that rat snake movement frequency was 0.07. We then averaged this value across all individuals of each species to calculate the proportion of snake detections in which a snake was moving for each week of the nesting season. Because snakes would move out of the range of the towers and activity data were not only evenly distributed seasonally, we used frequency of activity (defined as the no. of movements detected divided by the no. of observations). Also, because we were unable to determine a snake's exact location (~30 m accuracy) using the ARUs, we could not reliably estimate the distance moved per day using ARUs. Because most studies are limited to manual radiotelemetry, we compared the activity patterns generated by manual and automated radiotelemetry to understand how any differences could influence results from other studies. We used linear regression to explore relationships between the two activity estimates (distance moved per day from manual radiotelemetry and movement frequency from ARUs) for each snake species.

### Nest monitoring and predator identification

To explore daily nest survival in relation to snake activity, we found and monitored nests in 2011, 2012, and 2013. We focused mostly on abundant species whose nests were low enough to monitor with a mirror pole and to attach our cameras to without climbing (~2.5–3 m: Table 1). We located nests by searching available nesting habitat and using behavioral cues. Additionally, we used 15 miniature cameras (Cox et al. 2012a) to film a number of the monitored nests. As we were setting up cameras, we disguised them with vegetation and attached them to shrub and tree limbs 0.5–1 m from nests. To decrease the risk of abandonment, we placed cameras only at nests that had progressed beyond the laying stage. We checked nests every 2 days and considered nests successful if they fledged one or more nestlings or depredated if nestlings vanished more than 2 days before the estimated fledging date. We excluded the last monitoring interval of nests if we were uncertain of the fate (for those without cameras). After a nest was preyed on, we reviewed the video to identify the predator species.

**Table 1:** Bird nests monitored (*N* = 463) and filmed (*N* = 202) at the Ellenton Bay Set Aside Research Area from 2011 to 2013

Species		No. of nests monitored	No. of nests filmed
Blue Grosbeak	*Passerina caerulea*	42	25
Brown Thrasher	*Toxostoma rufum*	53	27
Carolina Wren	*Thryothorus ludovicianus*	3	3
Common Yellowthroat	*Geothlypis trichas*	1	0
Eastern Towhee	*Pipilo erythrophthalmus*	1	1
Indigo Bunting	*Passerina cyanea*	42	19
Mourning Dove	*Zenaida macroura*	16	9
Northern Cardinal	*Cardinalis cardinalis*	257	85
Northern Mockingbird	*Mimus polyglottos*	7	7
Northern Parula	*Parula americana*	1	1
Orchard Oriole	*Icterus spurius*	1	1
Painted Bunting	*Passerina ciris*	11	7
Prairie Warbler	*Dendroica discolor*	1	0
Red‐eyed Vireo	*Vireo olivaceus*	1	0
Red‐winged Blackbird	*Agelaius phoeniceus*	1	0
Yellow‐billed Cuckoo	*Coccyzus americanus*	6	4
White‐eyed Vireo	*Vireo griseus*	18	16
Yellow‐breasted Chat	*Icteria virens*	1	1

We used logistic exposure (Shaffer 2004) with Proc GENMOD (SAS 9.2; SAS Inst., Cary, NC) to assess the influence of snake activity on daily nest survival. Models were developed using distance moved per day by rat snakes and racers, and movement frequency of rat snakes and racers. We also included models for day of year (a quadratic term of Julian date) and year to account for seasonal and annual variation independent of snake behavior. We ranked models using Akaike's information criterion (AICc). We assessed models for all combined shrub‐nesting species and then for each species with large enough samples to analyze separately (*N* = 4).

To assess the influence of snake behavior on species‐specific predation rates, we used multinomial logistic regression models with Proc GLIMMIX with a logit link function using data only from nests with known fates (filmed nests). Data were organized into 1‐day filming interval and then the corresponding “fate” of each nest at the intervals end. Fates consisted of predation by the snake species of interest (rat snake or racer, depending on the analysis), predation by a predator other than the snake species of interest, or survived. Nests that failed for reasons unrelated to predation were excluded from analyses. We evaluated support for each of the following models: weekly mean distance moved per day by rat snakes, weekly mean distance moved per day by racers, frequency of movement by rat snakes, and frequency of movement by racers. Additionally, we evaluated models for day of year (a quadratic term for Julian date) and year to account for seasonal and annual variation in predation risk. We present predator‐specific predation rates as the converse of daily survival rate (Shaffer 2004) or the number of failed nests due to the snake of interest divided by the number of exposure days for those nests.

## Results

### Snake activity

From May to July 2011 and March to July 2012 and 2013, we hand‐tracked rat snakes and racers. In total, we hand‐tracked 19 individual rat snakes and 11 racers and accumulated 1380 and 756 relocations, respectively. Additionally, we monitored snakes with automated radiotelemetry in 2012 and 2013. With automated radiotelemetry, we monitored 15 rat snakes and 7 racers and accumulated approximately 34,000 rat snake movements and 55,000 movements by racers.

**Figure 2:**
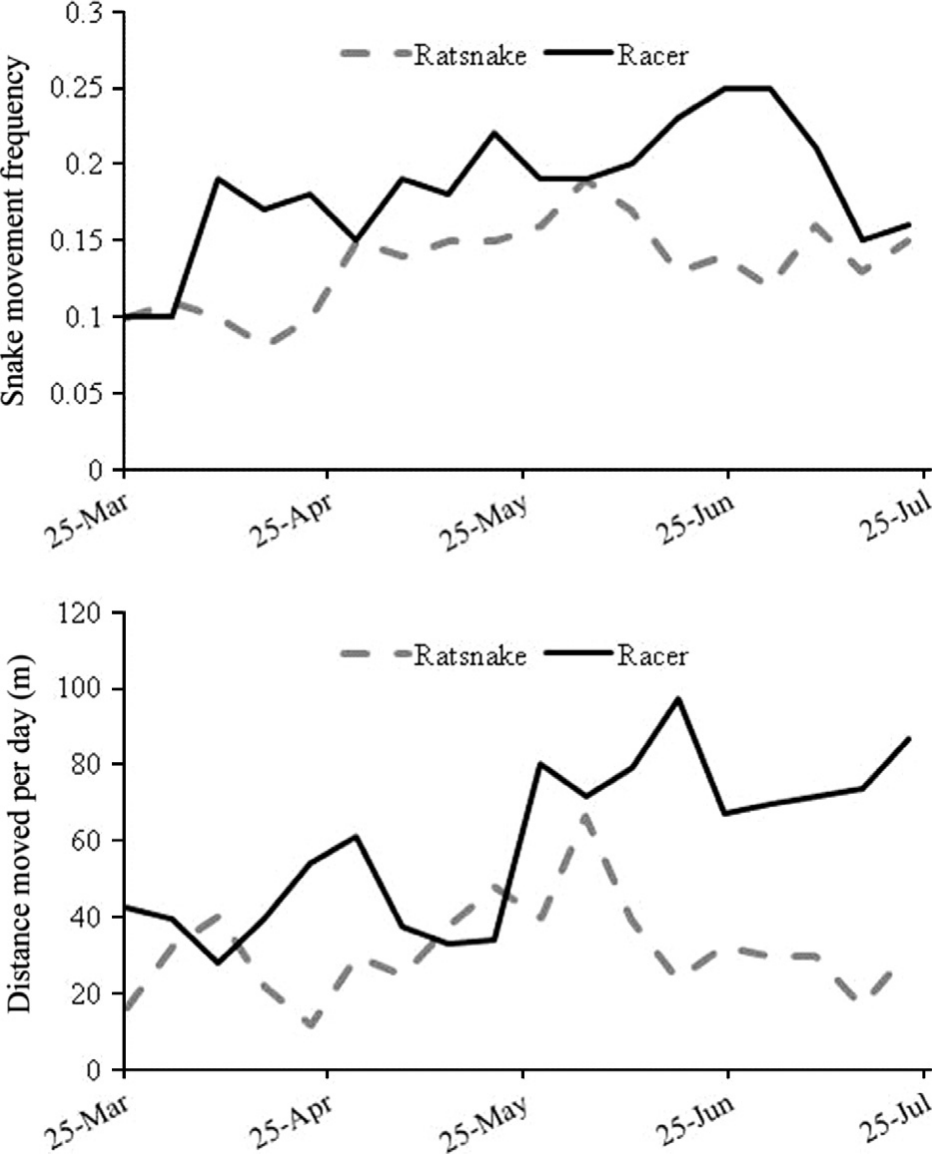
Distance moved per day and frequency of movement by 1‐week period for rat snakes (*Pantherophis obsoletus*) and racers (*Coluber constrictor*) tracked with manual and automated radiotelemetry at the Savannah River Site, South Carolina.

Rat snakes exhibited peaks in movement frequency and distance moved per day associated with dispersal from hibernacula (in late March) and mating (late May and early June: Fig. 2). Rat snakes were least active in March (10% activity) and most active in May and June (16%), with a decline in July (14%). Racers were least active early in the season (March: 10%) and were most frequently active in June (20%) and July (18%), generally increasing their activity across the summer. Rat snakes were not filmed preying on nests in March but preyed on six nests in April, 16 in May, 13 in June, and four in July. Racers did not prey on or visit nests during March or April but were filmed at 11 nests in May, nine in June, and three in July.

Distance moved per day and movement frequency of rat snakes were positively related (*r* ^2^ = 0.57, *P *<* *0.001). Both indices showed a peak of rat snake activity in May–June and the least activity occurring in the early spring. However, movement frequency was greater for rat snakes late in the summer, whereas distance moved per day decreased. The two activity indices were more weakly related for racers (*r* ^2^ = 0.17, *P *=* *0.071), although both indices showed a step‐wise increase in racer activity as the season progressed. Because the correlation between distance moved and activity frequency was moderate to weak for both species, we used both measures in analyses.

### Nest monitoring

We monitored a total of 463 nests of 17 bird species (Table 1) for a combined 5680 exposure days (mean DSR (SE): 0.799 ± 0.012). We collected enough data on four species to analyze them individually: northern cardinals (nests: 257, exposure days: 3306, DSR: 0.937 ± 0.006), blue grosbeaks (*Passerina caerulea*: nests: 42, exposure days: 559, DSR: 0.88 ± 0.013), brown thrashers (*Toxostoma rufum*: nests: 53, exposure days: 637, DSR: 0.851 ± 0.008), and indigo buntings (nests: 42, exposure days: 448, DSR: 0.842 ± 0.01). Of those, we filmed 206 nests for 3300 exposure days and identified predators for 137 nest predations. We filmed the nests of 13 species of birds (Table 1). The most frequently filmed nests were those of northern cardinals (nests: 85, exposure days: 1447), blue grosbeaks (nests: 25, exposure days: 376), brown thrashers (nests: 27, exposure days: 409), and indigo buntings (nests: 19, exposure days: 322). The most frequently observed predator was the rat snake, accounting for 38 nest predation events (28% of total). Racers were identified as predators at 17 bird nests (12% of total). Including rat snakes and racers, we identified at least 18 nest predators including corn snakes (*Pantherophis guttata*: 20), coachwhips (*Masticophis flagellum*: 5), American or fish crows (*Corvus brachyrhnchos* or *C. ossifragus*: 11), brown‐headed cowbirds (*Molothrus ater*: 11), blue jays (*Cyanocitta cristata*: 3), kites (*Elanoides forficatus*: 2), buteo hawks (4), accipiter hawks (3), eastern screech owls (*Otus asio*: 3), Carolina wrens (*Thyothorus lutovicianus*: 1), gray catbirds (*Dumetella carolinensis*: 2), at least two species of ants (*Solenopsis invicta* and *Chromatagaster spp*.: 8), raccoons (*Procyon lotor:* 8), and a bobcat (*Felis rufus:* 1) (DeGregorio et al. 2014a).

Nest predators differed between bird species (Table 2). Rat snakes were responsible for 35% of brown thrasher nest predations, whereas racers were never documented preying on brown thrasher nests. Blue grosbeak nests were often preyed on by racers (26% of nest predations) and avian predators (37%), likely because they often nested in open areas favored by racers and avian predators. Conversely, indigo buntings were infrequently preyed on by snakes (8% racers and 8% rat snakes) and instead were often preyed on by avian predators (50%). Cardinals were most frequently preyed on by avian predators (34%) and rat snakes (26%).

**Table 2:** Number of nest predation events of each focal nesting bird species attributed to each major nest predator or nest predator guild

	BLGR	BRTH	INBU	NOCA	All Species
Rat snake	3	8	1	14	38
Racer	5	0	1	7	17
Other Snake Species	2	9	2	8	25
Avian Predators	7	4	6	18	40
Mammals	0	0	1	2	9
Ants	2	2	1	4	8
Total Events	19	23	12	53	137

### Snake activity and daily nest survival

**Figure 3:**
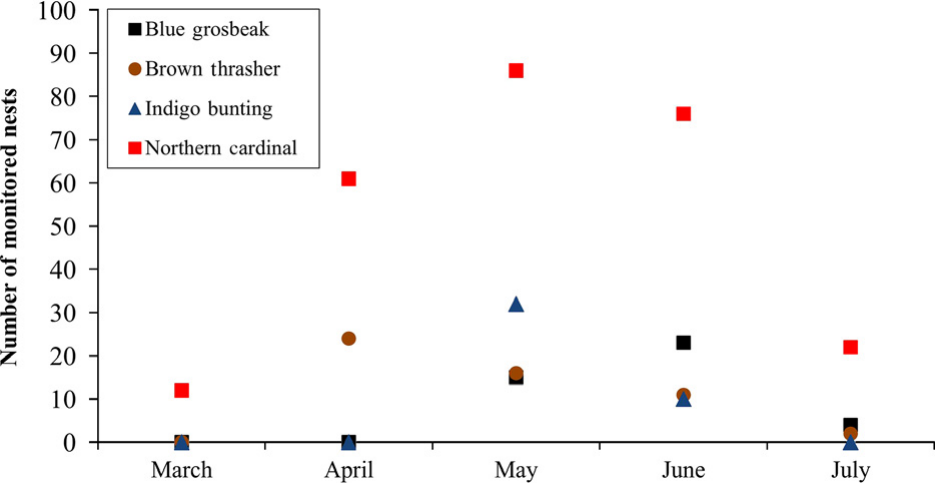
Number of nests of each focal bird species found and monitored by month.

We found and monitored relatively few nests in March. Most avian nesting at our site occurred between April and June (~90% of all monitored nests: Fig. 3). Thus, although we measured activity for snakes early in the season, most avian nest survival analyses were restricted to narrower timeframes.

**Figure 4:**
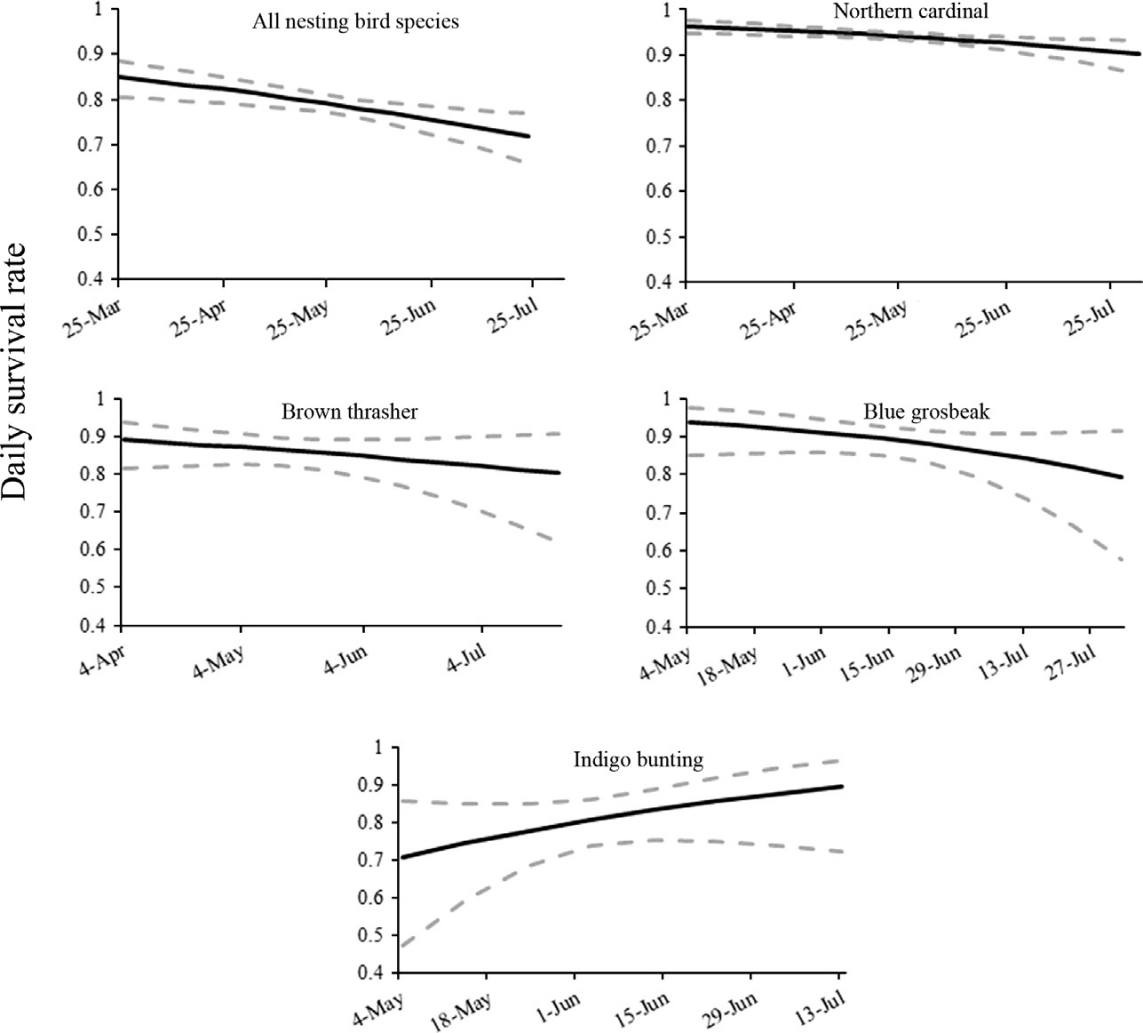
Seasonal variation in daily nest survival rate for all birds and focal bird species Ellenton Bay Set Aside Research Area, South Carolina, during the 2011–2013 nesting seasons.

When all nests (i.e., not just nests at which predators were identified) of all shrub‐nesting species were combined, daily nest survival rate declined over the season (Fig. 4). Daily nest survival of blue grosbeaks, brown thrashers, and northern cardinals followed similar patterns. Indigo buntings were the only species that experienced higher survival late in the nesting season (Fig. 4). Daily nest survival was highest at the start of the nesting season when snakes were relatively inactive, but over the entire season, snake activity was at best weakly associated with nest predation. Day of year was the top‐ranked model explaining variation in avian nesting success when all bird species were combined (Table 3). Day of year accounted for 70% of the weight of evidence and was 4 delta AICc units away from the second ranked model (constant survival), which accounted for only 8% of the total weight of evidence. No other models accounted for >10% of the total weight of evidence. This pattern was most likely influenced by the most abundant species, the northern cardinal, for which nesting success was most influenced by day of year (weight of evidence = 94%). For brown thrashers and indigo buntings, the top‐ranked model influencing daily nest survival was year, although for each species, there was considerable model uncertainty. Both species had significant interannual variation in survival. For example, in 2013, brown thrasher nest success was nearly four times greater than it was in 2011 or 2012. Similarly, indigo bunting nest survival was nearly four times lower in 2011 than in 2012 or 2013. Despite this annual variation, trends in seasonal nest survival were similar between years. Finally, for blue grosbeaks, constant survival was the top‐ranked model and accounted for 23% of the total weight of evidence.

**Table 3:** Models accounting for variation in daily nest survival of 463 bird nests at the Ellenton Bay Set Aside Research Area, South Carolina 2011–2013. Frequency of movement and distance moved per day were calculated via manual and automated radiotelemetry for black racers (*Coluber constrictor*) and rat snakes (*Pantherophis obsoletus*), two nest predators on the site

	*K*	AIC	ΔAICc	Wi
All species				
Day of year	2	1697.39	0	0.70
Constant survival	1	1701.75	4.36	0.08
Racer distance	2	1702.43	5.04	0.06
Year	2	1702.97	5.58	0.04
Rat snake frequency	2	1702.97	5.58	0.04
Racer frequency	2	1703.20	5.81	0.03
Rat snake distance	2	1703.48	6.09	0.03
Blue Grosbeak				
Constant survival	1	174.76	0	0.23
Rat snake distance	2	175.05	0.29	0.20
Racer distance	2	175.10	0.34	0.20
Day of year	2	175.75	0.99	0.14
Year	2	177.04	2.28	0.07
Rat snake frequency	2	177.04	2.28	0.07
Racer frequency	2	177.04	2.28	0.07
Brown Thrasher				
Year	2	198.21	0	0.31
Rat snake frequency	2	198.21	0	0.31
Racer frequency	2	198.41	0.20	0.29
Constant survival	1	203.03	4.82	0.03
Day of year	2	204.53	6.32	0.01
Rat snake distance	2	205.20	6.98	0.01
Racer distance	2	205.25	7.04	0.01
Indigo Bunting				
Year	2	143.53	0	0.21
Rat snake frequency	2	143.53	0	0.21
Racer frequency	2	143.53	0	0.21
Constant survival	1	143.68	0.15	0.19
Rat snake distance	2	145.73	2.20	0.07
Day of year	2	145.77	2.25	0.07
Racer distance	2	146.11	2.58	0.06
Northern Cardinal				
Day of year	2	1020.60	0	0.94
Constant survival	1	1028.05	7.44	0.02
Racer distance	2	1029.75	9.15	0.01
Rat snake distance	2	1030.07	9.48	0.01
Year	2	1030.09	9.50	0.00
Rat snake frequency	2	1030.09	9.50	0.00
Racer frequency	2	1030.09	9.50	0.00

### Snake‐specific predation

In general, species‐specific predation rates by rat snakes and racers increased throughout the nesting season although there was substantial variation within the year, presumably due to local weather conditions. When analyzing only nests that were filmed, rat snake‐specific predation rates were greatest when rat snakes moved the longest distances (*F*_2,1539_ = 3.26, *P *=* *0.038). The likelihood of a nest being preyed on by a rat snake increased 2.8% for each unit increase in distance moved per day (β = 1.032, 85% CI: 1.029–1.035). Rat snake‐specific predation rate was not associated with rat snake movement frequency (*F*_2,1534_ = 0.14, *P *=* *0.874), day of year (*F*_2,1539_ = 1.89, *P *=* *0.152), or year (*F*_3,1537_ = 0.33, *P *=* *0.859). Racer‐specific nest predation was also mildly influenced by the mean distance moved per day by racers (*F*_2,1539_ = 2.85, *P *=* *0.058). However, predation by racers was not influenced by racer movement frequency (*F*_2,1534_ = 0.07, *P *=* *0.930), day of year (*F*_2,1539_ = 1.77, *P *=* *0.171), or year (*F*_3,1537_ = 0.00, *P *=* *0.998).

## Discussion

For birds at our study site, nest success declined over the nesting season, consistent with patterns observed elsewhere (e.g., Grant et al. 2005; Cox et al. 2012b; Borgmann et al. 2013). This trend may be affected by biotic (e.g., foliage phenology, individual quality) and abiotic (e.g., weather) factors, but we expected that this pattern would be linked to the activity of rat snakes and perhaps to racers. However, unlike previous studies (Sperry et al. 2008, 2012; Weatherhead et al. 2010), we found no evidence that the activity of rat snakes or racers influenced overall nest success at our site. These results were inconsistent with previously reported results. However, previous studies did not identify predators at nests, and thus, relationships between predator activity patterns and nest survival were inferred. When we restricted analyses to nests with known predator identities, however, we found that the distance moved per day by rat snakes and racers was linked to their respective nest predation rates. These results suggest that snakes at our study site find more nests when they (snakes) are most active. In the following discussion, we attempt to explain why our results differed from our predictions, reconcile our results with previous studies, and consider the broader implications of these patterns for nesting birds.

The lack of association between rat snake seasonal activity and nest survival was unexpected. Although rat snakes at our site exhibited seasonal activity patterns similar to those reported for other locations (Carfagno and Weatherhead 2008; Sperry et al. 2008, 2012), we did not detect an influence of snake activity patterns on overall nest survival for all birds studied or for individual species. Interestingly, rat snakes preyed on a similar proportion of nests at our site (28%) as has been reported for other regions where rat snake activity and nest survival were strongly linked (30% of black‐capped vireo nests: Stake and Cimprich 2003: 44% of golden‐cheeked warbler nests: Reidy et al. 2008). The lack of a clear relationship at our site may be due to the presence of a diverse predator community. We documented a minimum of 18 predator species at our site (DeGregorio et al. 2014a) and avian nest predators (raptors, corvids, and brown‐headed cowbirds) and other snake species (corn snakes, black racers, and coachwhips) collectively accounted for an equivalent proportion of nest predation as rat snakes. Predation rate by avian predators and other snakes often vary seasonally and differently by species (Benson et al. 2010; Cox et al. 2013), making it challenging to discern the effects of any single predator on overall trends.

Our data from filmed nests with known predators indicated that predation by rat snakes and racers was linked to their seasonal activity patterns (distance moved per day). Although the seasonal activity patterns of snakes may not drive seasonal patterns of nest survival, they do, as expected, explain species‐specific predation patterns. These results highlight the need to identify nest predators to understand the factors affecting patterns in nest survival (Benson et al. 2010). These data also support our prediction that snakes are most likely to encounter and prey on nests when they are most active.

Snake‐specific nest predation was better explained by distance moved per day by snakes, determined via manual radiotelemetry, than by snake movement frequency derived from automated radiotelemetry. This is the same index of snake activity that was used in previous studies of snake activity and patterns in nest survival (Sperry et al. 2008, 2012; Weatherhead et al. 2010). Although both indices are correlated, distance moved per day appears to be a better indicator of snake foraging behavior. Movement frequency may capture many small‐scale movements associated with basking or shuttling behavior rather than true movement across the landscape. Fortunately, manual radiotelemetry is the more common and accessible approach for researchers and is likely to provide a reliable indicator of broad seasonal snake movement associated with foraging.

If predation risk varies predictably through time, birds may nest more and invest more (larger or more eggs) during periods of low predation risk (Perrins 1970; Daan et al. 1990; Nager and van Noordwijk 1995; Borgmann et al. 2013). Recognition that snakes are important nest predators and that temperature has an important influence on their activity holds the potential for birds to have evolved nesting strategies driven by snake behavior (Weatherhead and Blouin‐Demers 2004). Although rat snakes appear to be important nest predators throughout their range (DeGregorio et al. 2014b), the association between their activity and nest predation may not be as strong as initially expected based on previous results (e.g., Sperry et al. 2008). It remains possible that where snakes account for a higher proportion of nest predation, snake‐specific adaptations by birds are possible. For example, in Texas and the midwestern United States, birds experience higher nest success early and later in the nesting season due to inactivity of their snake predators (Sperry et al. 2008; Weatherhead et al. 2010; Cox et al. 2013). In general, the more that a single species dominates local nest predation, the greater potential for birds to modify how they nest in ways specific to that predator. For temperate North American birds, however, camera studies suggest that a diverse suite of nest predators is the norm. Given that different predators may be active at different times and find nests in different ways, there is likely to be limited potential for birds to decrease their nest predation risk, which may explain why rates of nest predation are generally so high.

## Conflict of Interest

None declared.

## Supporting information


**Appendix S1.** Study Site Figure and Location of Nests and Automated Radiotelemetry Towers.
