# Supplementary figures and images for: Do seasonal patterns of rat snake (Pantherophis obsoletus) and black racer (Coluber constrictor) activity predict avian nest predation?
Source: Ecol Evol. 2016 Feb 26;6(7):2034–43. doi: 10.1002/ece3.1992 (PMC4831437; doi:10.1002/ece3.1992)

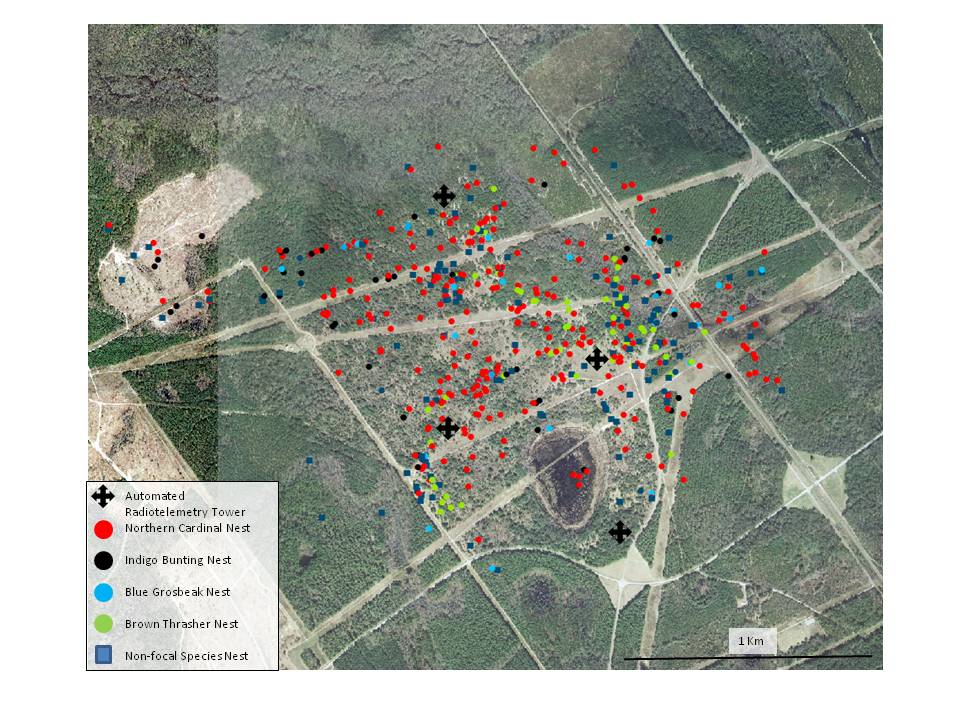

Supplement: Supplementary file 1 — Appendix S1. Study Site Figure and Location of Nests and Automated Radiotelemetry Towers. [file ECE3-6-2034-s001.jpg]
